# Association between hearing sensitivity and dopamine transporter availability in Parkinson’s disease

**DOI:** 10.1093/braincomms/fcad075

**Published:** 2023-03-21

**Authors:** Elena Garasto, Alessandro Stefani, Mariangela Pierantozzi, Rocco Cerroni, Matteo Conti, Simone Maranesi, Nicola B Mercuri, Agostino Chiaravalloti, Orazio Schillaci, Andrea Viziano, Arturo Moleti, Renata Sisto

**Affiliations:** Parkinson’s Center, Department of Systems Medicine, University of Rome ‘Tor Vergata’, Rome 00133, Italy; Parkinson’s Center, Department of Systems Medicine, University of Rome ‘Tor Vergata’, Rome 00133, Italy; Parkinson’s Center, Department of Systems Medicine, University of Rome ‘Tor Vergata’, Rome 00133, Italy; Parkinson’s Center, Department of Systems Medicine, University of Rome ‘Tor Vergata’, Rome 00133, Italy; Parkinson’s Center, Department of Systems Medicine, University of Rome ‘Tor Vergata’, Rome 00133, Italy; Parkinson’s Center, Department of Systems Medicine, University of Rome ‘Tor Vergata’, Rome 00133, Italy; Parkinson’s Center, Department of Systems Medicine, University of Rome ‘Tor Vergata’, Rome 00133, Italy; Department of Biomedicine and Prevention, University of Rome ‘Tor Vergata’, Rome 00133, Italy; Department of Biomedicine and Prevention, University of Rome ‘Tor Vergata’, Rome 00133, Italy; Department of Clinical Sciences and Translational Medicine, University of Rome ‘Tor Vergata’, Rome 00133, Italy; Department of Physics and NAST Centre, University of Rome ‘Tor Vergata’, Rome 00133, Italy; Department of Occupational and Environmental Medicine, Epidemiology and Hygiene, INAIL Research, Monte Porzio Catone (Rome) 00078, Italy

**Keywords:** Parkinson’s disease, DaTSCAN, basal ganglia nuclei, hearing loss, distortion product otoacoustic emissions

## Abstract

In a previous study, we observed: (i) significant hearing function impairment, assessed with pure tone audiometry and distortion product otoacoustic emissions, in patients with Parkinson’s disease, compared with a matched control group, and (ii) lateralization of the hearing dysfunction, worse on the side affected by more pronounced Parkinson’s disease motor symptoms. This study investigates the association between the basal ganglia dopamine transporter availability and the hearing function in Parkinson’s disease patients, focusing also on the lateralization of both dysfunctions, with respect to that of the motor symptoms, and introducing a further distinction between patients with left-sided and right-sided predominant motor symptoms. Patients with right-handed Parkinson’s disease with a recent estimation of ^123^I-FP-CIT striatal uptake were audiologically tested with pure tone audiometry and distortion product otoacoustic emissions. Thirty-nine patients were included in the study. A statistically significant association was found, in the left-side predominant group only, between the distortion product otoacoustic emission levels and the contralateral dopamine transporter availability, and between the hearing threshold and the dopamine transporter availability difference between the ipsi- and the contralateral sides. The hearing impairment lateralization correlated to the motor symptom asymmetry was found significant only in the left-side predominant patients. The association between hearing function and basal ganglia dopamine transporter availability supports the hypothesis that the peripheral hearing function decline associated with dopamine depletion is involved in Parkinson’s disease development, with a significant difference between patients with left- and right-sided predominant motor symptoms. These findings also suggest that peripheral hearing function evaluation and its lateralization could be key elements for subtyping the disease.

## Teaser

Garasto *et al*. deal with hearing dysfunction in Parkinson’s disease, and its association with dopamine transporter uptake in basal ganglia, reporting a laterality effect, mostly seen when distortion product otoacoustic emissions are examined in Parkinson’s disease patients with a prominent left-side motor deficit.

## Graphical Abstract

**Graphical abstract:**
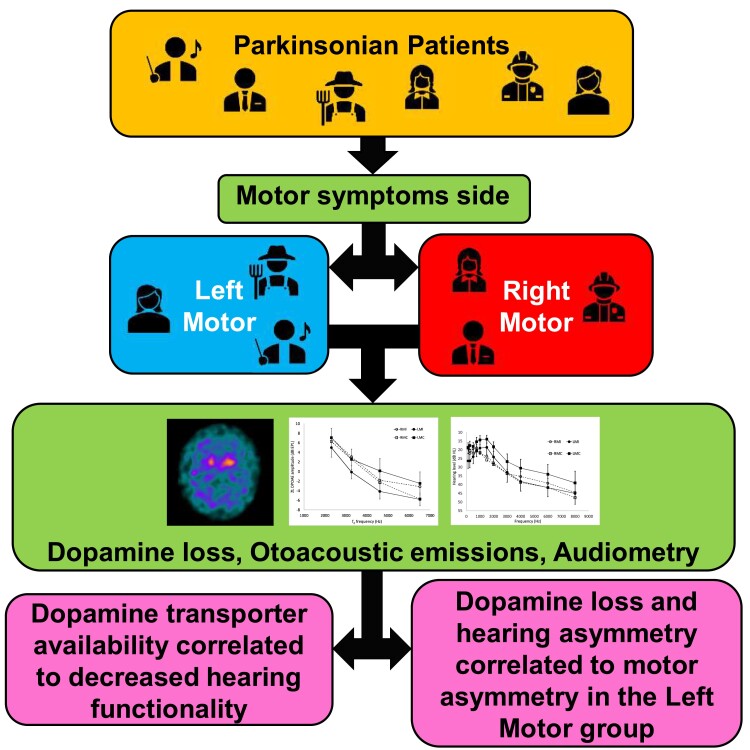

## Introduction

Motor dysfunction in Parkinson’s disease has been proved to be directly related to the progressive loss of nigrostriatal dopaminergic neurons.^1^ Furthermore, dopamine is a neurotransmitter involved in several functional systems, including the auditory network. The main dopaminergic neural population, involved in the auditory network, is located in the lateral olivocochlear bundle, which reaches the afferent auditory nerve, forming axodendritic synapses under the inner hair cells.^2,3^ It was hypothesized that dopamine acts as an inhibitor, decreasing cochlear response and, consequently, protecting the cochlea from the excess of excitotoxicity.^4,5^

An experimental study compared audiological measurements in wild-type mice and in mice with targeted deletions of dopamine receptors D1, D2, D4 and D5.^3^ With respect to the wild type, the D2 knock-out mice showed (i) reduced distortion product otoacoustic emission (DPOAE) and auditory brainstem response (ABR) levels and (ii) increased ABR thresholds. This experiment inspired the study by Pisani *et al*.,^6^ in which a group of *de novo* Parkinson’s disease patients were evaluated with audiological tests before and after the assumption of levodopa at therapeutic dose. The average DPOAE levels were found to be significantly increased after treatment. Therefore, it was hypothesized that patients before treatment could be considered similar to the D2 knock-out mouse model, with respect to the effect of dopamine on hearing function.

The idea of an essential role of dopamine in hearing function justifies the study of the auditory system in Parkinson’s disease patients. There is ample evidence of hearing impairment in Parkinson’s disease,^7-10^ leading to the view of hearing loss as a non-motor symptom of the disease, although the underlying pathophysiological mechanism has not been fully clarified. It was recently demonstrated that asymmetric hearing in Parkinson’s disease patients mirrors the asymmetry of the Parkinson’s disease motor symptoms.^11,12^

Here, we test the hypothesis that the impaired hearing functionality observed in Parkinson’s disease patients, and its asymmetry, are directly related to dopamine depletion. This hypothesis implies that a significant association should be observed between basal ganglia dopamine transporter (DAT) availability and audiological variables. Specifically, a strong association should be expected between hearing function on each side and the contralateral loss of nigrostriatal dopaminergic transport. Contralateral DAT binding would be expected to be positively associated with DPOAE but negatively associated with the hearing threshold.

Based on these premises, in the present clinical study, audiological variables of a group of patients with Parkinson’s disease were analysed and correlated with DAT uptake, measured via ^123^I-FP-CIT SPECT (DaTSCAN), in three subregions (putamen, caudate and striatum).

## Materials and methods

### Patient selection

A group of patients with a diagnosis of idiopathic Parkinson’s disease, according to Movement Disorder Society Clinical Diagnostic Criteria,^13,14^ were considered for enrolment in the study. Informed consent was given by the participants, in conformity with the Declaration of Helsinki. The procedures were approved by the local ethics committee at the University of Rome ‘Tor Vergata’ (protocol no. 7/18, 7 February 2018). Inclusion criteria required: (i) to have no history of otological/labyrinthine disorders; (ii) to have no concomitant neurological diseases, other than Parkinson’s disease, or abnormalities at the brain MRI, or cognitive impairment assessed by Mini-mental State Examination; (iii) to be right handed and (iv) to have appropriate DAT defects on the ^123^I-FP-CIT SPECT recently performed.

All patients who met the above criteria underwent neurological evaluation: Parkinson’s disease severity and progression were scored by Hoehn and Yahr stage; patients’ motor disabilities were quantified by the motor examination section of the Movement Disorder Society-Unified Parkinson’s Disease Rating Scale (MDS-UPDRS) Part III.

Patients maintained their regular treatment and were evaluated in ON state. A medical history interview was conducted to assess all external risk factors that could interfere with auditory dysfunction: chronic exposure to noise or chemical agents, use of potentially ototoxic drugs and high auditory risk at work. Patients with pathologies affecting the auditory system and/or audiological risk factors were excluded after the anamnestic interview.

All patients underwent DaTSCAN, and the ^123^I-FP-CIT uptake was measured at putamen (all 39 patients), caudate (37) and striatum (31). The average time interval from the DaTSCAN to the audiological evaluation was 18 months (max: 32 months, min: 5 months). Once the neurological examination was completed, all patients underwent audiological testing.

### Audiological testing

**Figure 1:**
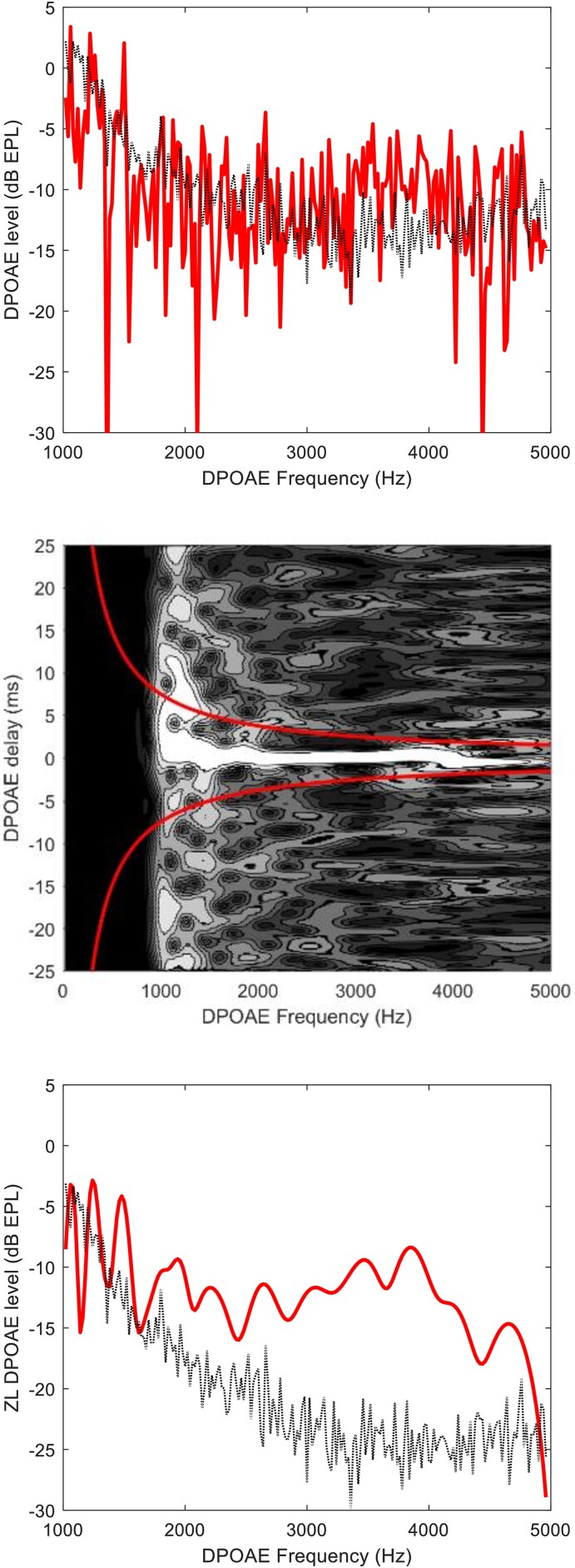
**Distortion product otoacoustic emission spectrum and time-frequency filtering.** Top: typical weak response of an elderly patient with Parkinson’s disease. Distortion product otoacoustic emission spectrum (solid line) and noise floor (dotted line), before time-frequency filtering. Middle: wavelet representation of the same response. Bottom: distortion product otoacoustic emission spectrum and noise floor after time-frequency filtering.

Audiometric hearing levels and DPOAE levels were measured in an audiometric booth, in the same session, in both ears. Pure tone audiometry (PTA) thresholds were evaluated at eleven standard audiometric frequencies from 0.125 to 8 kHz, down to −5 dB hearing level with a 5 dB accuracy. DPOAE sensitivity to sensorineural hearing loss is well-established.^15,16^ The nonlinear cochlea, stimulated at two nearby frequencies, *f*_1_ and *f*_2_, produces intermodulation distortion products at frequency *f*_DP_ = 2*f*_1_ − *f*_2,_^17^ clinically associated with frequency *f*_2_. DPOAE spectra were recorded with high frequency resolution (20 Hz) and were time-frequency filtered to unmix the distortion and reflection components.^18,19^ Focusing on the unmixed distortion (or zero-latency, ZL) component removes the uncertainty associated with DPOAE fine-structure amplitude fluctuations and lowers the noise level by up to 12 dB. This is shown in Fig. 1. In the unfiltered spectrum (top panel), signal-to-noise ratio barely exceeds unity only between 3 and 4 kHz. The wavelet representation shows (mid) that noise is distributed over the whole time-frequency plane, while the ZL DPOAE component is concentrated in a bright horizontally elongated spot within the filtering region, defined by the two red hyperbolas. After filtering (bottom), the signal-to-noise ratio is significantly improved. Such a low noise floor is necessary to measure the DPOAE response in elderly and/or hearing-impaired patients, and the usual averaging strategy cannot be used on some neurological patients, who cannot undergo tests involving a long recording time.

Ear-canal calibration of both the stimulus forward pressure and the otoacoustic emission response was performed, to reduce uncertainties associated with the probe insertion depth.^20^ The unmixed ZL components integrated over four half-octave bands, centred at *f*_2_ = 2305, 3260, 4610, 6520 Hz, were used as DPOAE outcome variables.

### DaTSCAN and ^123^I-FP-CIT uptake of putamen, caudate and striatum

DaTSCAN was performed, according to the methodology set up by our group with imaging acquired 4 h after 185 MBq (5 mCi) of ^123^I-FP-CIT injection. To prevent thyroid uptake of free radioactive iodide, perchlorate (1000 mg) was administered at least 30 min before.^21^ Parameters of SPECT acquisition, in accordance with Chang’s method,^22^ and construction of the standard region of interest (ROI) were described in previous studies.^23,24^ For each patient, the same ROIs were used and calculated as ^123^I-FP-CIT binding = (ROI − Occ)/Occ, where ROI indicates the mean count in the specific basal ganglia (putamen or caudate nucleus) and Occ represents the mean counts in the occipital cortex. For the striatum, the three adjacent transverse slices showing most intense uptake were considered; then, the mean specific to non-specific binding ratio was calculated.

### Data handling and statistical analysis

All statistical analyses were performed using the software R (version 4.2.0, R Foundation for Statistical Computing, Vienna, Austria). A significance criterion *P* < 0.05 was conventionally adopted.

### Multivariate mixed-effects models

Multivariate mixed-effect linear regression models were fitted to the data. In this approach, particularly useful in cases of non-independent measures performed on the same subject, the subject is considered as a random variable.

The ^123^I-FP-CIT uptake of putamen, caudate end striatum, ipsilateral and contralateral to the side of the worst motor symptoms was treated as continuous numeric variables (named ipsi and contra DAT). A DAT asymmetry variable was defined as the difference between the ipsi- and contralateral DAT. All DPOAE levels were treated as a unique variable, being in the half-octave frequency band, a four-level factor. In the PTA case, an 11-level factor was similarly introduced. Age was treated as a continuous variable.

A two-level factor was introduced and named ‘laterality’, distinguishing ipsi- and contralateral ears with respect to the body side that was more affected by motor symptoms. An additional two-level factor was added, the variable ‘side’, specifying if the side more affected by motor symptoms is the left (LM) or the right (RM) one.

As the mixed-effect models do not produce a determination coefficient, the statistical significance of the model itself was tested by fitting two models, one including the fixed effect factor of interest and the other one without it. The two models are compared by means of an ANOVA test.

### DaTSCAN

In order to investigate the association between the DAT availability and the hearing function variables, in the case of both DPOAE and PTA, mixed-effect linear regression models were used.

The six fitted models are:


(1) $$\begin{array}{rl} \mathrm{Mod} & =\;\mathrm{lme}(\mathrm{AUDIO}∼\mathrm{side}+\mathrm{age}+\mathrm{DAT}\\ & +\mathrm{frequency},\mathrm{random}=∼1|\mathrm{subject}) \end{array}$$

where AUDIO was either the ZL DPOAE level or the PTA hearing level, and DAT was that measured at the putamen, caudate or striatum. We started with a general model containing the ipsi, contra and difference DAT. An iterative procedure selected the significant variables in Equation (1).

Terms up to two-factor interaction were considered. The effect of the interaction term side:DAT was analysed, to evaluate differences between the RM and LM subgroups.

### Statistical association with clinical variables

Multivariate mixed-effect models were also used to test the statistical significance of a set of clinical variables: levodopa equivalent daily dose (LEDD), disease duration and staging and motor impairment score. Clinical and demographic data of the enrolled patients are summarized in Table 1.

**Table 1:** Clinical and demographic average data for Parkinson’s disease patients

	ALL	LM	RM
Number of subjects	39	17	22
Sex	21 M; 18 F	10 M; 7 F	11 M; 11 F
Age (years)	63	64	63
Disease duration (months)	36	35	39
Hoehn and Yahr score	1.6	1.6	1.7
MDS-UPDRS Part III	17	17	17
LEDD (mg)	250	239	259

## Results

### Study population

A total of 39 consecutive outpatients with Parkinson’s disease were included in the study (18 females, 21 males, average age = 63 years, max = 81 years, min = 45 years). All subjects reported lateralized predominant motor symptoms, clinically detectable. Twenty-nine of them had already been included in the data set of a previously published study:^11^ they were also included in this present study since they were all right-handed and had undergone DaTSCAN in line with the present study design. In 45% of the enrolled patients, the left-hand side was more affected by the Parkinson’s disease motor symptoms (side = LM: 17 patients, side = RM: 22 patients).

Classification according to age, sex, disease duration (months), staging (Hoehn and Yahr score), motor impairment (MDS-UPDRS Part III), laterality as predominant motor side (right or left) and concomitant therapy, evaluated by LEDD, is shown in Table 1.

### Statistical association with clinical variables

No significant association was found between the clinical variables (disease duration, staging, motor impairment score and LEDD) and the audiological variables. No statistically significant difference between the LM and RM groups was found in the clinical variables.

### DaTSCAN

**Figure 2:**
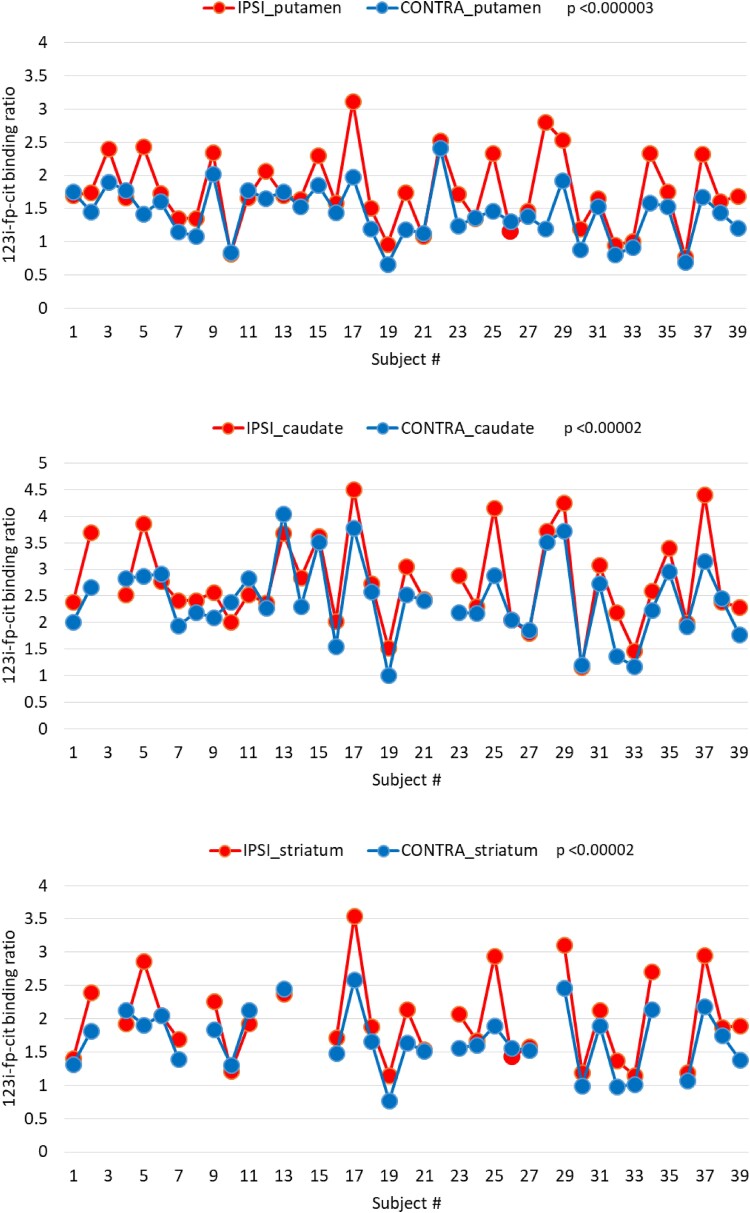
**Distribution of ipsilateral and contralateral dopamine transporter availability.** Systematically lower dopamine transporter availability is measured on the brain side contralateral to that of the worst motor symptoms, for all three basal ganglia, compared with the ipsilateral side, using a paired *t*-test. Note that the large inter-subject fluctuations are correlated to each other for the three basal ganglia.

The distributions relative to the contralateral DAT for the ipsi and contra sides (more and less affected by the Parkinson’s disease motor symptoms) are reported in Fig. 2. The expected lateralization effect is well visible, with the DAT systematically lower on the contralateral side with respect to that more affected by the motor symptoms. A paired *t*-test shows significant differences between the ipsi and contra DAT for the striatum, caudate and putamen, with *P* < 0.00002, *P* < 0.00002 and *P* < 0.000003, respectively. This expected result is reported mainly to confirm the reliability of the DAT data set.

### Association between DPOAE and DAT

#### Principal effects

The regressions between the DPOAE amplitude and the DAT binding are significant for all the basal nuclei. Fitting model (1) with contralateral DAT, the comparison between the model containing the fixed-effect ‘contralateral DAT’ and the reduced model, which does not contain it, is statistically significant for putamen, caudate and striatum (*P* < 0.02, 0.01, 0.03, respectively). The regression coefficients are also similar for putamen, caudate and striatum (*β* = 2.7, 2.6, 3.0, respectively), meaning that a DPOAE level decrease of nearly 3 dB is expected for a DAT decrease of one unit.

As expected, the effects of age and frequency were found significant. The effect of the variable side (being more affected by motor symptoms on the left or right hand side, LM versus RM) was not significant at the principal level.

#### Two-factor interaction effects

The interaction term of the variable side with all contralateral DATs was found significant. The slope of the regression between the ZLDP and the DAT was found significantly steeper in the LM case. For the LM, at the putamen caudate and striatum, respectively, *β* = 4.5, 3.4, 4.8, with *P* < 0.01, 0.002, 0.003, while for the RM: *β* = 1.5, 2.5, 2.0, with *P* = n.s., <0.03, n.s.

### Association between hearing threshold and DaTSCAN

#### Principal effects

The only significant regression was that with the difference between ipsi- and contralateral caudate DAT availability in the case of the caudate. The regression coefficient is *β* = 2.4, with *P* < 0.003. The larger the DAT asymmetry, the worse the hearing threshold.

#### Two-factor interaction effects

The interaction term of the variable side with DAT was also found significant for the PTA hearing level. Positive correlation with the DAT uptake asymmetry was found in the LM group only, with, for caudate, striatum and putamen, respectively: *β* = 4.9, 5.7, 9.1, with *P* < 0.0001, *P* < 0.0001, *P* < 0.0001.

### Laterality effects in Parkinson’s disease patients (revisited, introducing LM versus RM)

**Figure 3:**
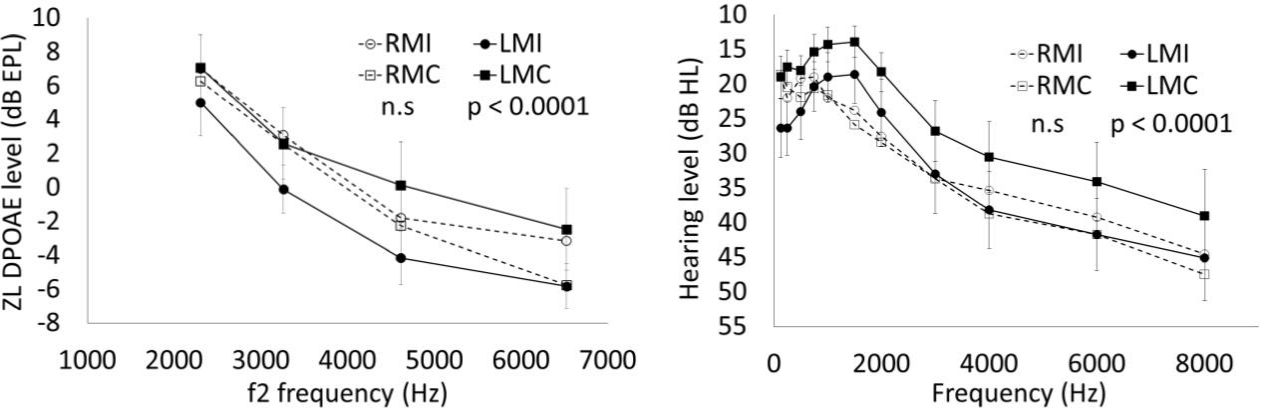
**Laterality effect.** For both Distortion product otoacoustic emission levels (left) and audiometric hearing levels (right), the difference between contralateral and ipsilateral hearing function is significant only in the LM subgroup, using a mixed-effect linear regression model. Error bars display standard errors (shown only for the LM subgroup, for plot readability). LMC, contralateral ears of left-sided predominant motor symptom patients; LMI, ipsilateral ears of left-sided predominant motor symptom patients; RMC, contralateral ears of right-sided predominant motor symptom patients; RMI, ipsilateral ears of right-sided predominant motor symptom patients.

Figure 3 shows the average DPOAE response (left) and PTA hearing level (right) as functions of frequency, in four groups of ears, the ipsilateral ears of LM and RM patients (LMI and RMI, obviously being right and left, respectively) and their contralateral ears (LMC and RMC, respectively).

The comparison between the model containing the fixed-effect ‘laterality’ and the reduced model, which does not contain it, gave a statistically significant result for the ZLDP (*P* < 0.02). The regression coefficient for the linear mixed-effect model was *β* = −1.5, *P* < 0.02, i.e. on average, the DPOAE response of the ipsilateral ear (i.e. that of the side that was more affected by the motor symptoms) is lower by 1.5 dB than that of the contralateral ear. The effect of ‘laterality’ becomes much larger if the subsample of LM subjects is selected. In this case, *β* = −3.0, *P* < 0.0001, while the regression was not significant for the RM subgroup. This finding confirms the lateralization effect (*β* = −1.99) observed in a previous study,^11^ restricting it, in an amplified form, to the LM subgroup.

The same comparison was statistically significant also for the PTA hearing level (*P* < 0.01). The regression coefficient is *β* = 2.1, *P* < 0.01, meaning that an average increase of the hearing threshold of 2.1 dB is observed on the side ipsilateral to the motor symptoms. As in the case of ZLDP, the data set was divided into LM and RM patients. The effect of laterality in the LM subgroup was very large: *β* = 6.5, *P* < 0.0001, while the regression coefficient for the RM subgroup was not statistically significant.

For clarity, all the statistically significant results are summarized in Table 2, separately for the three subregions, for the whole sample and for the LM and RM subgroups.

**Table 2:** Summary of the statistically significant results

Result	ALL	LM	RM
DAT availability is lower on the side contralateral to motor symptoms	SPC	SPC	SPC
DAT availability is correlated to DPOAE level	SPC	SPC	–C
DAT availability asymmetry is correlated to audiometric level	–C	SPC	
DPOAE level is lower (worse) on the side ipsilateral to motor symptoms	YES	YES	NO
Hearing level is higher (worse) on the side ipsilateral to motor symptoms	YES	YES	NO

The first three rows refer to correlations between DAT availability and audiological variables, and the last two rows refer to the lateralization of the hearing damage on the same side of the motor symptoms. C, Caudate; P, Putamen; S, striatum.

## Discussion

Results from this study seem to support our hypothesis, based on previous work,^3,6^ that cochlear impairment in Parkinson’s disease might share a common mechanism of dopaminergic depletion with the disease itself. Results also emphasize the role of the auditory system as a non-motor symptom of Parkinson’s disease. This is not the first study to investigate the relation between non-motor symptoms and striatal DAT availability: olfactory dysfunction and mild cognitive impairment have also been associated with nigrostriatal dopaminergic loss.^25,26^ However, this is the first study to look at its association with audiological variables assessed by PTA and DPOAEs, further suggesting a possible lateralization effect of cochlear dysfunction, mirroring the course of asymmetric motor impairment.

The association between DPOAE and DAT striatal uptake was found to be significant for both brain sides. The strength of such an association was much higher when looking at the results of the basal ganglia DAT uptake contralateral to the most affected motor symptoms. This last result is consistent with previous results,^11,12^ corroborating the evidence that DPOAE responses are significantly worse in the ear ipsilateral to the side manifesting worse motor symptoms, as shown in Sisto *et al*.^11^ In more specific terms, hearing dysfunction in Parkinson’s disease patients seems to parallel the asymmetry of motor impairment. These results may lead to the hypothesis that, in the pathogenesis of Parkinson’s disease, not only motor symptoms but also non-motor symptoms can develop asymmetrically, especially when considering bilateral sensory symptoms. For instance, Nolano *et al*.^27^ demonstrated a lower intraepidermal nerve fibre density in the side more affected by motor symptoms in a Parkinson’s disease population, investigated through skin biopsy.

There are multiple putative links between the auditory system and degenerative processes within Parkinson’s disease. Firstly, Lewy pathology (accumulation of misfolded α-synuclein) or dopaminergic neuronal degeneration might be involved in Parkinson’s disease–related auditory dysfunction. Indeed, α-, β- and γ-synuclein had been localized in the mammalian cochlea,^28^ with the first one predominantly present at the base of outer and, albeit in less amount, inner hair cells, thus suggesting a possible role in the neurodegenerative process of the inner ear in Parkinson’s disease. Secondly, dopaminergic and glutamatergic neurotransmission in the inner ear, with the former having a protecting role on excitotoxicity,^4,5^ seem to mirror the synaptic interplay seen in the basal ganglia. Previous experience in a small cohort of de-novo patients suggested a beneficial dopamine-mediated effect on DPOAE levels.^6^ However, we did not find clear correlation with LEDD, both here and in previous study,^11^ possibly because the increase of LEDD may balance disease progression. Additionally, it has been speculated that compensatory mechanisms of auditory stimuli processing might occur when peripheral hearing dysfunction increases or disease progresses.^7^ In this regard, future studies could be focused on better defining these aspects, investigating, e.g. the effect on auditory variables of deep brain stimulation or acute intake of dopaminergic treatment, as it has been observed for other non-motor symptoms.^29,30^ The findings of this study are different between DPOAE and PTA. In fact, in the LM group only, a significant association was found between hearing threshold and the difference between DaTSCAN data from the ipsi- and contralateral caudate nuclei, with worse hearing threshold in the subjects with a large asymmetry of DAT availability in the caudate nuclei. The difference between the DAT availability associations with PTA and DPOAEs can be due to the fact that patients’ alertness and collaboration play a relevant role in the audiometric outcome, whereas DPOAEs are objectively sensitive to peripheral function and, as hypothesized here, to the dopaminergic cochlear pathways.

Another aspect that emerges in this study is the stronger association of audiological measurement and DAT availability in the LM subgroup of patients: when dividing the data set into the LM and RM patient subgroups, the association between hearing threshold and the ipsi-/contralateral difference in the caudate nuclei or the striatum was found to be significant only in the case of LM patients. Furthermore, considering DPOAEs, the effect of ‘laterality’ becomes more significant when a subsample of LM subjects is selected.

In this scenario, it is worth noting that several studies have evaluated differences in motor and non-motor symptoms between Parkinson’s disease patients who experience worse impairment on the left side of the body versus those more affected on the right side. In a recent study,^31^ left side lateralization was associated with faster symptom progression and worse outcomes in multiple clinical domains. Cubo *et al*.^32^ also associated left-predominant disease with worse motor and non-motor performance. A study by Haaxma *et al*.^33^ attributed greater motor dysfunction of the more affected hand in Parkinson’s disease patients to higher predominantly right-hemisferic dopamine depletion. Finally, Fiorenzato *et al*.,^34^ by evaluating asymmetrical DaTSCAN uptake in the putaminal region, showed that asymmetric dopaminergic neuronal loss influenced Parkinson’s disease cognitive and motor performance, as well as disease progression: prevalent loss in the right hemisphere (e.g. worse motor performance on the left side) was associated with greater symptom severity, whereas left hemisphere denervation seemed to affect cognitive symptoms. These studies provide a possible explanation for the results obtained in the present study when an audiological outcome, such as PTA, is used. Indeed, PTA performance may be affected by higher cortical functions other than the auditory pathway. With regard to non-motor involvement, Zhu *et al*.^35^ observed that Parkinson’s disease patients with left-dominant symptoms experienced a greater impairment of sleep quality than patients with right-dominant symptoms. Some authors speculated that a possible explanation for these differences may reside in an increased dopaminergic reserve in the dominant left hemisphere for individuals who are right handed. Further research is needed with respect to the possible asymmetrical presentation of other non-motor Parkinson’s disease features.

Possible limitations of this study are related to the small number of patients. Furthermore, the association with motor impairment was fully investigated, due to the fact that patients were not evaluated during the off-medication stage.

To our knowledge, this is the first study in which a significant and lateralized association between DAT availability and hearing loss is found. The evidence of an association between Parkinson’s disease and hearing loss is rapidly growing in the literature, but no evidence existed so far to the effect that audiological variables could be quantitatively related to an objective measurement of disease such as dopamine neuronal ending depletion in the basal ganglia. The association between DAT availability and hearing function, and their correlated lateralization suggest that the asymmetric development of cochlear dysfunction and that of motor impairment share a common source.

## Data Availability

All the data related to this study may be made available upon written request, from Parkinson’s Center, Department of Systems Medicine, University of Rome ‘Tor Vergata’, in accordance with the European data-protection legislation (General Data Protection Regulation).

## Abbreviations

ABR =: auditory brainstem response
DAT =: dopamine transporter
DPOAE =: distortion product otoacoustic emission
LEDD =: levodopa equivalent daily dose
LM =: left-sided predominant motor symptom
MDS-UPDRS =: Movement Disorder Society-Unified Parkinson’s Disease Rating Scale
PTA =: pure tone audiometry
RM =: right-sided predominant motor symptom
ROI =: region of interest
ZL =: zero-latency
